# Supplementary material for: Predicting cell types with supervised contrastive learning on cells and their types
Source: Sci Rep. 2024 Jan 3;14:430. doi: 10.1038/s41598-023-50185-2 (PMC10764802; doi:10.1038/s41598-023-50185-2)
Supplement: Supplementary file 1 — Supplementary Figure S1. [file 41598_2023_50185_MOESM1_ESM.pdf]

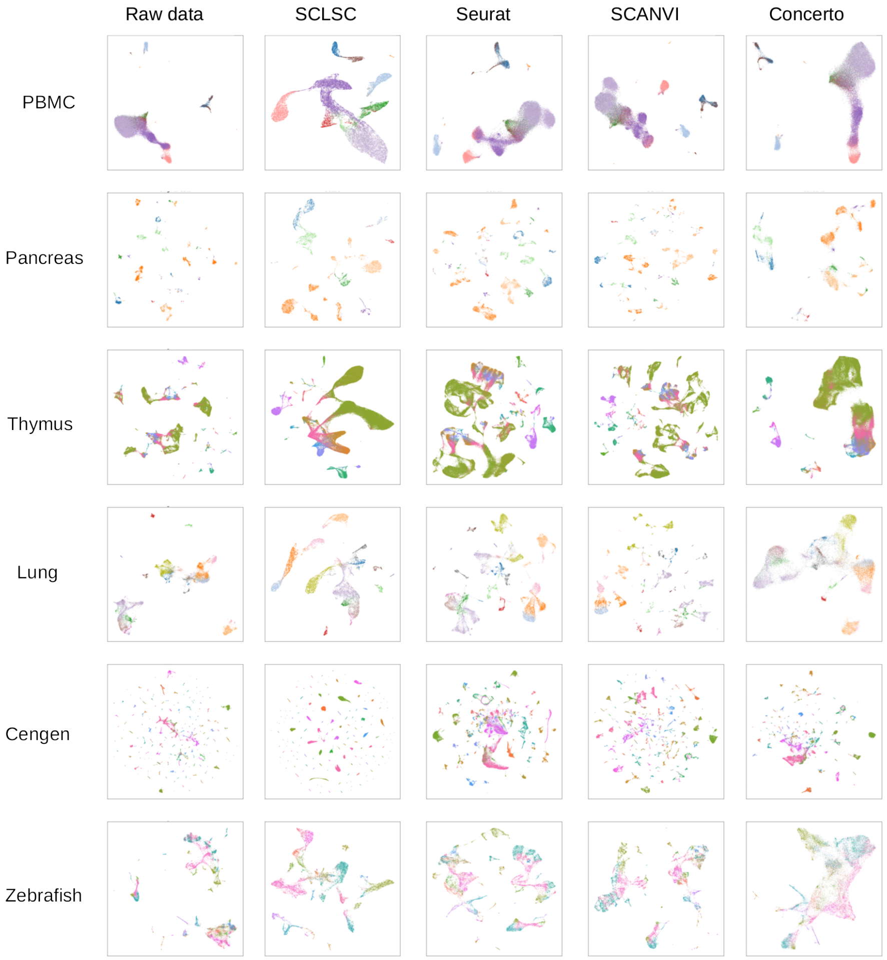

Figure S1: **The UMAP visualization of benchmark datasets**. SCLSC utilizes cell type representations to guide cells towards proximity to their respective cell type representations while simultaneously moving away from representations of other cell types. This process encourages cells of the same type to cluster together and separate from cells of different types, thereby facilitating more straightforward cell label predictions as shown in the visualization.
